# Revolutionizing Drug Delivery: The Impact of Advanced Materials Science and Technology on Precision Medicine

**DOI:** 10.3390/pharmaceutics17030375

**Published:** 2025-03-15

**Authors:** Mohamed El-Tanani, Shakta Mani Satyam, Syed Arman Rabbani, Yahia El-Tanani, Alaa A. A. Aljabali, Ibrahim Al Faouri, Abdul Rehman

**Affiliations:** 1 RAK College of Pharmacy, Ras Al Khaimah Medical and Health Sciences University, Ras Al Khaimah P.O. Box 11172, United Arab Emirates; arman@rakmhsu.ac.ae; 2 Department of Pharmacology, RAK College of Medical Sciences, Ras Al Khaimah Medical and Health Sciences University, Ras Al Khaimah P.O. Box 11172, United Arab Emirates; 3 Royal Cornwall Hospital NHS Trust, Truro TR1 3LJ, UK; y.el-tanani@nhs.net; 4 Department of Pharmaceutics and Pharmaceutical Technology, Faculty of Pharmacy, Yarmouk University, Irbid 21163, Jordan; alaaj@yu.edu.jo; 5 RAK College of Nursing, Ras Al Khaimah Medical and Health Sciences University, Ras Al Khaimah P.O. Box 11172, United Arab Emirates; faouri@rakmhsu.ac.ae; 6 Department of Pathology, RAK College of Medical Sciences, Ras Al Khaimah Medical and Health Sciences University, Ras Al Khaimah P.O. Box 11172, United Arab Emirates; rehman@rakmhsu.ac.ae

**Keywords:** advanced drug delivery systems, precision medicine, nanocarriers, bio responsive polymers, artificial intelligence in therapeutics

## Abstract

Recent progress in material science has led to the development of new drug delivery systems that go beyond the conventional approaches and offer greater accuracy and convenience in the application of therapeutic agents. This review discusses the evolutionary role of nanocarriers, hydrogels, and bioresponsive polymers that offer enhanced drug release, target accuracy, and bioavailability. Oncology, chronic disease management, and vaccine delivery are some of the applications explored in this paper to show how these materials improve the therapeutic results, counteract multidrug resistance, and allow for sustained and localized treatments. The review also discusses the translational barriers of bringing advanced materials into the clinical setting, which include issues of biocompatibility, scalability, and regulatory approval. Methods to overcome these challenges include surface modifications to reduce immunogenicity, scalable production methods such as microfluidics, and the harmonization of regulatory systems. In addition, the convergence of artificial intelligence (AI) and machine learning (ML) is opening new frontiers in material science and personalized medicine. These technologies allow for predictive modeling and real-time adjustments to optimize drug delivery to the needs of individual patients. The use of advanced materials can also be applied to rare and underserved diseases; thus, new strategies in gene therapy, orphan drugs development, and global vaccine distribution may offer new hopes for millions of patients.

## 1. Introduction

Over the last few decades, drug delivery systems have evolved rapidly because of the increasing complexity of drugs and growing need for precision medicine [1,2,3,4,5]. As pharmaceuticals advance, the limitations of current delivery methods become more apparent. These conventional approaches are very effective in certain cases, but they are not very useful in the modern therapeutic area because they have poor bioavailability, are systemically toxic and cannot target specific tissues. These shortcomings underscore the urgent need for innovative strategies capable of delivering drugs with greater precision, minimized side effects, and enhanced therapeutic efficacy. The integration of cutting-edge materials has played a pivotal role in the evolution of drug delivery technologies, facilitating the development of innovative solutions that address the shortcomings of conventional methods. To address this, advancements in material science have introduced functional, adaptable, and biocompatible systems, revolutionizing drug delivery efficiency [6,7]. The integration of cutting-edge materials has played a pivotal role in the evolution of drug delivery technologies, facilitating the development of innovative solutions that address the shortcomings of conventional methods. Among these innovations, nanocarriers, hydrogels, and bioresponsive polymers have garnered significant attention for their ability to enhance the stability, precision, and effectiveness of therapeutic agents [8,9,10]. Nanocarriers, such as liposomes, dendrimers, and nanoparticles, are capable of targeting specific cells or tissues with remarkable accuracy, minimizing side effects and improving the therapeutic index [11]. Their small size and structural versatility enable them to encapsulate and transport a wide range of small-molecule drugs and biologics, ensuring improved treatment efficacy and controlled release at the intended site of action.

Hydrogels, another breakthrough in controlled drug delivery, are three-dimensional, hydrophilic polymeric networks that provide a platform for minimally invasive applications while enabling the sustained release of drugs at the target site over time [12]. This characteristic makes hydrogels particularly valuable in tissue engineering and regenerative medicine, where controlled drug release is critical for therapeutic success [13]. Furthermore, bioresponsive polymers have revolutionized drug delivery by responding to environmental stimuli, such as pH, temperature, or enzymatic activity, to trigger site-specific drug release [14,15]. These polymers ensure that therapeutic agents are activated only under specific physiological conditions, enhancing both safety and efficacy.

In addition to macroscopic hydrogels, microgels and nanogels have emerged as promising stimuli-responsive drug delivery platforms [16]. These crosslinked polymeric networks exhibit the unique ability to swell or shrink in response to external stimuli, such as temperature, pH, ionic strength, or specific biomolecular interactions, making them highly adaptable for targeted and controlled drug release. Unlike traditional polymeric nanoparticles, the nanogel architecture plays a critical role in modulating interactions with biological environments, influencing circulation time, cellular uptake, and biodistribution [17].

Nanogels, with their nanoscale size (typically 20–200 nm), provide enhanced permeability and retention (EPR) effects, allowing them to accumulate in tumor tissues and inflamed regions, improving passive targeting efficiency [18]. The high-water content of nanogels makes them biocompatible, while their soft, deformable structure allows for easy diffusion through biological barriers [19]. Their stimuli-responsive behavior can be finely tuned by modifying polymer composition, ensuring on-demand drug release in response to microenvironmental changes. For instance, pH-sensitive nanogels composed of poly(N-isopropylacrylamide) (PNIPAM) or poly(acrylic acid) (PAA) undergo conformational changes in acidic tumor environments, triggering the release of chemotherapeutic agents precisely at the tumor site [20].

Similarly, temperature-sensitive microgels have been designed to collapse or expand based on physiological temperature fluctuations, allowing for sustained and pulsatile drug delivery [21]. These materials are particularly useful in injectable hydrogel-based formulations, where temperature-triggered gelation ensures localized and prolonged drug retention at the target site. Additionally, enzyme-responsive nanogels, developed using biodegradable polymers such as chitosan, gelatin, or dextran, allow for targeted drug release in the presence of disease-specific enzymes, further improving therapeutic precision [22].

Given their superior biocompatibility, tunable degradation rates, and responsiveness to multiple stimuli, microgels and nanogels represent a significant advancement in smart drug delivery systems, offering novel solutions for cancer therapy, regenerative medicine, and inflammation-targeted treatments. Their ability to integrate with bioresponsive polymers further expands their applicability, positioning them as next-generation carriers in precision medicine.

By incorporating microgels and nanogels alongside nanocarriers, hydrogels, and bioresponsive polymers, drug delivery systems can achieve greater flexibility, enhanced targeting, and more controlled therapeutic effects, opening new avenues for advanced and personalized treatment strategies.

These advanced materials also play a critical role in addressing some of the most significant challenges in cancer treatment, including drug resistance. In oncology, nanocarriers and functionalized drug delivery systems have improved the targeted delivery of chemotherapeutics to tumor cells, while minimizing damage to normal tissues [23,24]. In a similar vein, sustained-release systems have transformed the management of chronic diseases such as diabetes, cardiovascular diseases, and neurological disorders by reducing the frequency of drug administration, providing continuous therapeutic effects, and improving patient compliance [25,26,27]. In the field of vaccine delivery, advanced materials such as lipid nanoparticles have been pivotal, particularly in the rapid development of mRNA vaccines, as demonstrated during the COVID-19 pandemic [28,29]. These advancements in drug delivery technology are not only enhancing the effectiveness of treatments but also expanding the possibilities for addressing previously unmet medical needs.

Despite their excellent potential, the usage of advanced material-based drug delivery systems is limited by several translational issues. The biocompatibility and long-term safety of these materials remains a critical concern. For example, some nanomaterials may induce an immune response or accumulate in the body and cause toxicity [30,31]. Furthermore, the large-scale production of these sophisticated systems poses challenges in batch-to-batch consistency, quality control, and cost efficiency. The need for specialized infrastructure further restricts their scalability. Additionally, regulatory frameworks for nanoparticle-based therapeutics remain underdeveloped, as most of these materials represent entirely new classes of therapeutic systems with limited precedent in clinical applications. Collaborative efforts between researchers, industry stakeholders, and regulatory bodies are essential to establish clear approval pathways and standardized guidelines for their clinical translation.

A critical yet often overlooked factor in nanocarrier-based drug delivery is degradability within the human body, which directly influences drug release kinetics, biodistribution, and clearance mechanisms. The degradation rate of nanoparticles must be carefully tailored to match therapeutic requirements, ensuring that drugs are released in a controlled manner, while minimizing toxic accumulation of residual nanomaterials. The byproducts of nanoparticle degradation and their interactions with cellular metabolism, immune response, and organ clearance pathways play a crucial role in determining long-term safety and efficacy.

For instance, polymeric nanoparticles such as PLGA (polylactic-co-glycolic acid) degrade into lactic and glycolic acid, which are metabolized via natural biochemical pathways and excreted safely. However, inorganic nanoparticles, such as gold or silica-based systems, may persist in tissues for extended periods, leading to potential bioaccumulation risks. Researchers are addressing this issue by developing biodegradable metallic nanoparticles and hybrid organic-inorganic nanocarriers that undergo controlled degradation through enzymatic or pH-sensitive mechanisms.

Additionally, nanoparticle degradability must align with the intended therapeutic window—a system that degrades too quickly may result in premature drug release and reduced efficacy, while one that degrades too slowly could prolong exposure and increase toxicity risks. Understanding how nanocarrier degradation products interact with intracellular metabolic pathways is essential for predicting toxicity profiles, optimizing dosing regimens, and enhancing biocompatibility.

The future of nanocarrier design is being revolutionized by artificial intelligence (AI) and machine learning (ML), which can accelerate the development of advanced drug delivery systems by analyzing massive datasets and identifying optimal material properties [32,33,34]. AI can play a transformative role in predicting and optimizing nanoparticle degradation profiles by

▪ Simulating nanoparticle breakdown in physiological conditions, ensuring ideal drug release kinetics and clearance rates.
▪ Identifying degradation pathways that produce biocompatible byproducts, reducing toxicity risks.
▪ Customizing nanocarrier formulations based on patient-specific metabolism and disease states, contributing to precision medicine.
▪ Optimizing material composition to enhance biodegradability without compromising stability and drug-loading efficiency.

Furthermore, AI-driven computational models can integrate pharmacokinetic (PK) and pharmacodynamic (PD) data to fine-tune nanoparticle design for maximal therapeutic benefit. This aligns with the broader goals of personalized medicine, where treatment regimens are tailored to a patient’s genetic, physiological, and environmental characteristics.

With advances in nanotechnology, AI-driven material optimization, and precision drug delivery, researchers are now designing next-generation biodegradable nanocarriers that degrade in a programmed, controlled manner, ensuring optimal therapeutic action and minimal toxicity. These advancements expand treatment possibilities for rare diseases, chronic conditions, and personalized therapies, while addressing long-standing challenges of nanocarrier stability, safety, and scalability.

By integrating degradation kinetics, metabolic compatibility, and AI-driven optimization, nanomedicine is evolving toward safer, more effective, and personalized treatment strategies, unlocking new possibilities in clinical translation and regulatory acceptance.

Advanced material science is paving the way for the future of drug delivery, with innovations set to transform the pharmaceutical landscape by enhancing treatment precision and reducing side effects. The ability of these materials to improve targeting accuracy, minimize adverse effects, and optimize patient outcomes highlights their revolutionary potential. As research continues to overcome translational barriers and expand applications, these advanced materials are poised to become an integral part of modern medicine.

This review aims to provide a comprehensive insight into these advancements, aiming to inspire further innovation and interdisciplinary collaboration in this rapidly evolving field. The convergence of material science, biotechnology, and clinical medicine holds the key to unlocking the full potential of advanced drug delivery systems, ultimately expanding treatment options and improving patient care worldwide.

## 2. Emerging Materials in Drug Delivery

The development of innovative materials has significantly advanced drug delivery systems, addressing the limitations of conventional approaches. These materials provide precise control over drug release, targeted distribution, and bioavailability, effectively overcoming key challenges in modern therapeutics. By enabling drug delivery tailored to specific physiological needs, these advanced materials have paved the way for safer and more effective treatments across a wide range of diseases.

This section explores three major categories of emerging materials in drug delivery: nanomaterials, hydrogels, and bioresponsive polymers. These cutting-edge technologies have transformed pharmaceutical science by introducing novel therapeutic strategies and redefining the way we approach treatment.

### 2.1. Nanomaterials

Nanomaterials are now central to the solution of drug delivery problems, offering precise targeting, reduced systemic toxicity and improved therapeutic outcomes [5]. These nanoscale materials possess unique physicochemical properties, such as small size (typically 1–100 nm), high surface area-to-volume ratio, tunable surface chemistry, and the ability to encapsulate diverse therapeutic agents, ranging from small molecules to complex biologics [35]. Furthermore, nanomaterials enhance pharmacokinetics and biodistribution, addressing challenges such as rapid clearance, poor solubility, and non-specific targeting [36].

Among the earliest nanocarriers, liposomes are phospholipid bilayer vesicles that can encapsulate both hydrophilic (within the aqueous core) and hydrophobic (within the lipid bilayer) drugs. Over time, the modification of liposomes with polyethylene glycol (PEG), cholesterol, and ligand-based targeting moieties has led to increased stability, prolonged circulation half-life, and improved targeting efficiency [37]. For example, Doxil^®^, a pegylated liposomal formulation of doxorubicin (liposome size ~100 nm, surface-modified with PEG for steric stabilization), has set the benchmark in oncology by reducing cardiotoxicity and enhancing therapeutic efficacy [38,39]. The next generation of active-targeted liposomes, functionalized with antibodies (e.g., trastuzumab for HER2+ breast cancer) or small-molecule ligands, enables highly specific drug delivery to cancer cells, reducing off-target toxicity [40,41,42].

Dendrimers, another class of nanoscale polymeric carrier, are highly branched, monodisperse macromolecules with precise molecular weight, well-defined shape, and tunable surface functionalization. These properties allow the conjugation of therapeutic agents, imaging probes, and targeting ligands for multifunctional drug delivery systems [43,44]. Typically, dendrimers such as poly(amidoamine) (PAMAM) dendrimers (size 2–10 nm, positively charged surface) have been explored for cancer therapy, gene delivery, and antimicrobial applications due to their high drug-loading capacity and ability to cross biological barriers [45,46]. Additionally, PAMAM dendrimers functionalized with polyethylene glycol (PEG) and targeting ligands have shown promise in enhancing cellular uptake and minimizing cytotoxicity [47,48,49].

Engineered polymeric nanoparticles, particularly those composed of polylactic-co-glycolic acid (PLGA), polycaprolactone (PCL), and chitosan, have been extensively utilized for controlled drug release and targeted delivery [50,51]. PLGA nanoparticles (50–200 nm), approved by the FDA for biomedical applications, offer biodegradability, biocompatibility, and tunable drug release profiles [52]. The surface functionalization of PLGA nanoparticles with PEG (PEGylation) improves systemic circulation, while the conjugation of ligands such as folic acid, transferrin, and aptamers enhances tumor-targeting efficiency. Chitosan nanoparticles (size ~100–300 nm, positively charged surface) are widely used for mucoadhesive drug delivery in ocular, nasal, and gastrointestinal systems due to their biocompatibility, bioadhesiveness, and ability to enhance drug permeability.

Among inorganic nanomaterials, metallic nanoparticles (gold, silver, and iron oxide) have demonstrated exceptional theranostic potential. Gold nanoparticles (AuNPs), available in tunable sizes (1–150 nm), exhibit strong surface plasmon resonance, enabling applications in photothermal therapy and imaging [53]. The surface modification of AuNPs with thiol-based ligands, antibodies, or polyethylene glycol (PEG) enhances stability and biocompatibility, making them valuable tools for targeted drug delivery and the photothermal ablation of tumors. Similarly, silver nanoparticles (AgNPs, typically 5–50 nm) exhibit broad-spectrum antimicrobial activity, disrupting bacterial cell membranes and generating reactive oxygen species (ROS) that induce oxidative stress [54]. These nanoparticles have been integrated into wound dressings, coatings for medical implants, and antimicrobial textiles to prevent infections [55,56].

In diagnostic applications, metallic nanoparticles are widely employed in point-of-care (POC) biosensors. For example, gold nanorods and nanostars, due to their tunable optical properties, have been incorporated into surface-enhanced Raman spectroscopy (SERS)-based diagnostic assays for the ultra-sensitive detection of biomarkers [57]. Additionally, superparamagnetic iron oxide nanoparticles (SPIONs, size 10–50 nm) have been utilized in magnetic resonance imaging (MRI) as contrast agents, offering real-time tracking of drug distribution and disease progression [58].

For theranostic applications, metallic nanoparticles functionalized with tumor-targeting ligands selectively accumulate in tumor microenvironments, facilitating dual-mode therapy and imaging. Gold nanoshells and nanocages, with their strong near-infrared (NIR) absorption, are widely used in photothermal therapy (PTT), where localized heat generation leads to selective tumor ablation while sparing surrounding healthy tissues [59]. Furthermore, hybrid nanoparticle systems, combining metallic and polymeric materials, offer synergistic advantages in controlled drug release, multimodal imaging, and combination therapy applications [60,61].

Among nano-enabled drug delivery systems, self-assembled polymeric micelles (size ~10–100 nm) formed from amphiphilic block copolymers have gained interest for hydrophobic drug solubilization and tumor-targeted delivery [62]. These micelles, commonly composed of PEG-PLA, PEG-PCL, or PEG-PGA copolymers, improve drug stability, prolong circulation time, and enhance bioavailability. The incorporation of targeting ligands (e.g., folate, transferrin) further improves tumor-selective uptake, minimizing systemic toxicity [63].

Nanocarriers such as liposomes, dendrimers, polymeric nanoparticles, and metallic nanoparticles offer diverse material properties, tunable sizes, and functional surface modifications, making them highly versatile for targeted drug delivery, controlled release, molecular imaging, and theranostic applications. The continuous advancements in nanomaterial engineering, surface functionalization, and hybrid nanoparticle platforms are driving next-generation precision medicine strategies, enabling enhanced therapeutic efficacy and personalized treatment approaches.

### 2.2. Hydrogels

Hydrogels are three-dimensional polymeric networks which can take up a large amount of water and are used as an effective medium for drug delivery. They are also biocompatible, their properties can be tuned, and they can mimic natural tissues; that is why they are so useful for the controlled and localized drug release.

#### 2.2.1. Injectable Hydrogels

Injectable hydrogels, known for their minimally invasive nature and ability to adapt to complex anatomical structures, have garnered significant recognition for their applications [64,65]. These hydrogels can be injected as a liquid and then form at the site of the body where they are needed, thus providing continuous release of the drug at the site [66]. This is particularly useful in the management of chronic diseases such as arthritis and cancer, where steady drug administration is required. Researchers are also attempting to develop injectable hydrogels as tissue engineering applications, which combine drug delivery and regenerative medicine [67,68,69,70].

#### 2.2.2. Responsive Hydrogels

The scope of their application has been further widened by the development of intelligent hydrogels that respond to certain stimuli. These stimulus-responsive hydrogels are developed to release the drug in response to some specific physiological cues such as pH, temperature, or enzyme activity [71,72]. For example, pH-dependent hydrogels can release chemotherapeutic agents in acidic environments, allowing for targeted delivery directly to tumors. Similarly, temperature-sensitive hydrogels, which respond to the temperature of the tumor site, remain in a liquid form at normal body temperature and solidify at higher temperatures, offering a controlled and prolonged drug delivery system [73]. These hydrogels are also being explored for injectable applications to enhance therapeutic efficacy.

### 2.3. Bioresponsive Polymers

**Figure 1:**
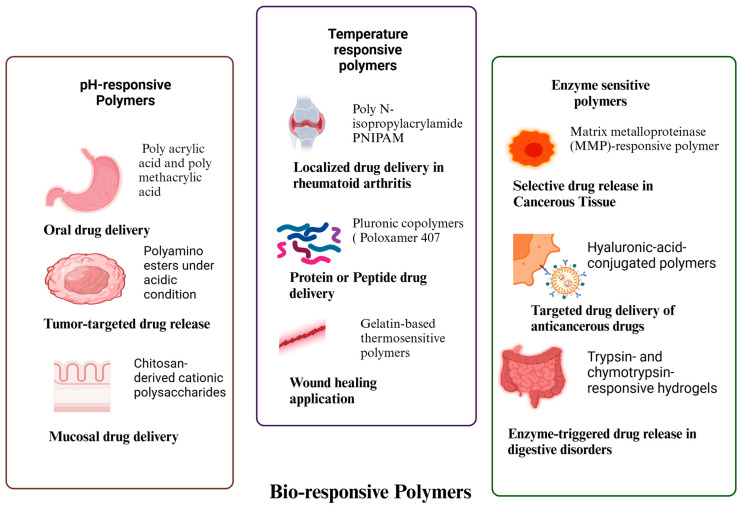
Bioresponsive polymers for drug delivery applications. This schematic illustrates the diverse applications of bioresponsive polymers in drug delivery, categorized by their triggering mechanisms: pH-responsive, temperature-responsive, and enzyme-sensitive. Examples of specific polymers and their applications are provided, showcasing their potential in various therapeutic areas, including oral drug delivery, tumor targeting, wound healing, arthritis treatment, cancer therapy, and digestive disorders.

Adaptive drug delivery systems based on bioresponsive polymers are advanced materials engineered to alter their properties in response to external or internal stimuli, enabling real-time drug release with enhanced precision and efficacy. By leveraging physiological triggers such as pH, temperature, and enzyme activity, these smart polymers ensure that therapeutic agents are selectively activated in diseased tissues, minimizing systemic exposure and off-target effects (Figure 1).

#### 2.3.1. pH-Responsive Polymers

pH-sensitive polymers are designed to exploit the slightly acidic microenvironment of tumor tissues, inflamed sites, or endosomal compartments, ensuring site-specific drug release while reducing systemic toxicity. These polymers typically contain acid-labile or ionizable functional groups, such as carboxyl (-COOH), amine (-NH2), or imidazole groups, which undergo protonation or deprotonation in response to pH fluctuations.

▪ Poly(acrylic acid) (PAA) and poly(methacrylic acid) (PMAA) exhibit pH-dependent swelling, making them ideal for oral drug delivery systems that bypass gastric degradation and release drugs in the intestines.
▪ Poly(β-amino esters) (PBAEs) and poly(N-vinyl imidazole) (PVI) degrade under acidic conditions, facilitating tumor-targeted drug release.
▪ Chitosan, a naturally derived cationic polysaccharide, remains soluble in acidic environments but forms a gel at physiological pH, making it suitable for mucosal and gastrointestinal drug delivery.

This strategy has proven particularly valuable in cancer therapy, where the tumor microenvironment (pH ~6.5–6.8) can trigger localized drug release, reducing off-target toxicity and improving therapeutic efficacy [74].

#### 2.3.2. Temperature-Responsive Polymers

Temperature-sensitive polymers undergo sol-gel phase transitions in response to physiological temperature fluctuations, making them highly effective for injectable and implantable drug delivery systems. These polymers are often thermosensitive hydrogels, which remain liquid at room temperature but form a gel upon injection into the body, creating a sustained drug release depot at the target site.

▪ Poly(N-isopropylacrylamide) (PNIPAM) is a well-known thermosensitive polymer that exhibits a lower critical solution temperature (LCST), around 32–35 °C, making it ideal for localized drug delivery in rheumatoid arthritis and postoperative pain management.
▪ Pluronic^®^ block copolymers (e.g., Poloxamer 407), composed of poly(ethylene oxide)-poly(propylene oxide)-poly(ethylene oxide) (PEO-PPO-PEO), form hydrogels at body temperature, making them suitable for sustained protein or peptide delivery.
▪ Gelatin-based thermosensitive hydrogels, crosslinked with genipin or transglutaminase, have been explored for biodegradable tissue scaffolds and wound healing applications.

These systems enhance drug retention at the injection site, allowing for localized therapy with prolonged effects, which is particularly beneficial for chronic inflammatory diseases such as rheumatoid arthritis [75,76].

#### 2.3.3. Enzyme-Responsive Polymers

Enzyme-sensitive polymers are specifically engineered to respond to the overexpression of disease-associated enzymes, triggering site-specific drug release only in pathological tissues. These polymers typically incorporate cleavable peptide sequences, ester bonds, or disulphide linkages, which are degraded by target enzymes, ensuring highly localized treatment with reduced systemic toxicity.

▪ Matrix metalloproteinase (MMP)-responsive polymers, such as PEGylated gelatin or polycaprolactone-based hydrogels, are degraded by MMP-2 and MMP-9, enzymes that are overexpressed in tumors and inflammatory sites, leading to selective drug release in cancerous tissues.
▪ Hyaluronidase-sensitive nanocarriers, composed of hyaluronic acid (HA)-conjugated polymers, degrade in response to tumor-associated hyaluronidase, facilitating targeted delivery of anticancer drugs.
▪ Trypsin- and chymotrypsin-responsive hydrogels, based on peptide crosslinked dextran or poly(ethylene glycol) (PEG), have been designed for enzyme-triggered drug release in digestive disorders.

These enzyme-responsive materials provide high specificity and precise activation, ensuring that therapeutic agents are released only in disease-affected regions, thereby minimizing unintended side effects [22,77].

Bioresponsive polymers represent a transformative advancement in drug delivery, enabling stimuli-triggered, highly localized, and controlled drug release. By leveraging pH, temperature, and enzyme-responsive mechanisms, these smart materials significantly enhance therapeutic precision, minimize systemic exposure, and improve patient outcomes. The continued development of next-generation bioresponsive polymers with optimized degradation kinetics, enhanced biocompatibility, and personalized adaptability is expected to revolutionize the landscape of targeted drug delivery [78].

### 2.4. Integration and Future Prospects

These advanced materials have already demonstrated their transformative potential in both clinical and preclinical settings when incorporated into drug delivery systems. Nanomaterials, hydrogels, and bio responsive polymers have introduced innovative approaches to drug delivery, paving the way for personalized and precision medicine. However, critical challenges, such as scalability, regulatory approval, and long-term biocompatibility, must be addressed to facilitate the smooth translation of these innovations from the laboratory to clinical application. Artificial intelligence and machine learning are expected to play a crucial role in material design and clinical outcome prediction (see below). Furthermore, ongoing research in biodegradable materials and environmentally friendly processing technologies will be essential for the sustainable development of advanced drug delivery systems. As these materials continue to evolve, they are set to provide new, innovative solutions to unmet medical needs, significantly enhancing patient care on a global scale.

## 3. Clinical Applications and Case Studies

The clinical applications of advanced materials in drug delivery have transformed therapeutic strategies in terms of addressing key challenges and enabling unprecedented precision and efficiency. These materials have shown remarkable versatility, and their impact is evident in oncology, chronic disease management, and vaccine delivery. Through the use of advanced platforms, such as nanocarriers, hydrogels, and polymer-based systems, researchers and clinicians are overcoming the limitations of traditional therapies. This section offers an additional look at these transformative applications.

### 3.1. Oncology 

Cancer treatment has for a long time been associated with the lack of specificity in drug delivery, which results in systemic toxicity and reduced therapeutic efficacy. New materials have resolved these problems, providing new approaches for the delivery of chemotherapeutics and immunotherapeutics with higher precision.

#### 3.1.1. Nanocarrier-Based Chemotherapy

**Figure 2:**
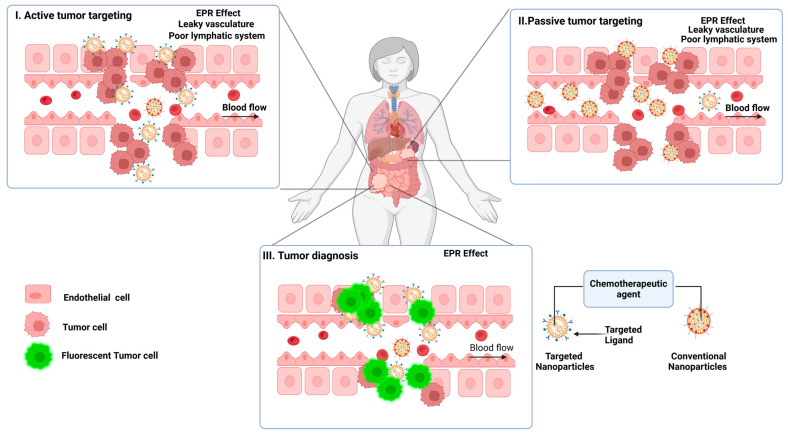
Tumor-targeting strategies using nanoparticles. This schematic illustrates three distinct approaches to tumor targeting using nanoparticles: (**I**) active tumor targeting, where nanoparticles are functionalized with ligands for specific tumor cell recognition; (**II**) passive tumor targeting, relying on the Enhanced Permeability and Retention (EPR) effect due to leaky vasculature and poor lymphatic drainage in tumor tissues; and (**III**) tumor diagnosis, utilizing fluorescent nanoparticles for imaging and detection. The figure highlights the key mechanisms involved in each strategy, including blood flow, nanoparticle accumulation, and tumor cell interactions.

Nanocarriers, including liposomes, dendrimers, and polymeric nanoparticles, have significantly enhanced the delivery of chemotherapy drugs, improving their therapeutic outcomes while minimizing systemic side effects [79]. Liposomal formulations, such as Doxil^®^, utilize the Enhanced Permeability and Retention (EPR) effect, a phenomenon wherein nanoparticles preferentially accumulate in tumor tissues due to the leaky vasculature and impaired lymphatic drainage characteristic of tumors [80,81,82]. This targeted drug accumulation in cancer cells not only reduces the amount of chemotherapy drug reaching normal tissues but also intensifies the drug’s effect at the tumor site, leading to higher therapeutic efficacy, with reduced side effects. The unique architecture of dendrimers allows for the controlled release of these drugs, maintaining optimal drug levels in the tumor over extended periods [83,84,85]. These dendrimers can be designed to carry a variety of drugs, including small molecules, proteins, and nucleic acids, enabling a personalized approach to treating complex cancers [86,87]. The controlled release mechanism of dendrimers ensures that the drugs are released in a sustained manner, reducing the need for frequent administration and improving treatment adherence. Polymeric nanoparticles also offer similar advantages, providing a versatile platform for the delivery of a wide range of therapeutic agents, enhancing drug stability, bioavailability, and targeting precision [88]. Together, these nanocarriers offer a promising approach to overcoming the limitations of conventional chemotherapy by ensuring targeted, controlled, and sustained drug release, ultimately improving patient outcomes (Figure 2).

#### 3.1.2. Multifunctional Platforms

The development of multifunctional platforms is an exciting advancement in personalized cancer therapy, combining both diagnostic and therapeutic capabilities within a single nanoparticle system. One of the most notable examples is the use of gold nanoparticles that are functionalized with both chemotherapeutic agents and imaging probes, enabling dual-mode therapy and monitoring [89]. These multifunctional nanoparticles can simultaneously deliver chemotherapy drugs to the targeted tumor cells and enable real-time monitoring of the treatment’s progress through imaging techniques such as CT (computed tomography) or MRI (magnetic resonance imaging). This dual functionality allows clinicians to visualize and assess the accumulation of the nanoparticles at the tumor site, ensuring that the drug is being delivered precisely where it is needed [90,91]. This monitoring capability also provides valuable information regarding the treatment’s effectiveness, enabling healthcare providers to make informed decisions about adjusting the therapy if necessary.

In addition to metallic nanoparticles, microbubbles have emerged as a highly promising theranostic platform due to their ultrasound-responsive drug release properties and imaging capabilities [92]. These gas-filled microscale carriers, typically composed of lipid, polymer, or protein shells, can serve as effective ultrasound contrast agents, while also enabling targeted and controlled drug delivery.

One of the key advantages of microbubble-based drug delivery is the ability to trigger drug release using ultrasound waves. Upon exposure to focused ultrasound pulses, microbubbles undergo cavitation, causing localized membrane disruption and enhanced drug penetration into targeted tissues. This strategy significantly improves drug bioavailability, ensuring that therapeutic agents are delivered precisely to diseased sites, such as tumors, inflamed tissues, or ischemic regions. This spatiotemporal control over drug release reduces systemic toxicity and enhances treatment efficacy.

Additionally, butyl cyanoacrylate-based microbubbles have demonstrated exceptional potential as ultrasound contrast agents, allowing for high-resolution imaging of blood flow, tumor vasculature, and organ perfusion [93]. Their biodegradable and non-toxic composition makes them particularly suitable for clinical applications, as they naturally degrade after imaging, minimizing long-term accumulation risks. Due to their dual functionality as both drug carriers and imaging agents, microbubbles represent a powerful theranostic tool, integrating real-time monitoring with targeted therapy to optimize patient outcomes.

The integration of diagnostic and therapeutic functions in microbubble-based platforms, alongside metallic nanoparticles, allows for a more personalized approach to cancer treatment, where therapy can be tailored to the patient’s specific tumor characteristics. This innovative strategy optimizes drug delivery, reduces off-target toxicity, and enhances treatment precision, ultimately contributing to more effective, individualized cancer care. By harnessing the unique capabilities of microbubbles for ultrasound-triggered drug release and high-resolution imaging, researchers are expanding the frontiers of theranostics, paving the way for next-generation, real-time precision medicine strategies.

#### 3.1.3. Overcoming Multidrug Resistance (MDR)

Multidrug resistance (MDR) remains one of the most significant challenges in cancer treatment, as it limits the effectiveness of chemotherapy and often leads to treatment failure. MDR occurs when cancer cells develop resistance to multiple, structurally unrelated chemotherapy drugs, often through mechanisms such as drug efflux, alteration of drug targets, or enhanced DNA repair. To overcome MDR, novel nanocarriers, such as polymeric micelles and exosome-mimicking nanoparticles, are being developed [94,95]. These systems offer advanced strategies to overcome the barriers posed by resistance mechanisms. Polymeric micelles are self-assembled nanostructures that can encapsulate hydrophobic chemotherapy drugs, protecting them from degradation in the bloodstream and ensuring their stability and sustained release at the tumor site [96]. These micelles can be engineered to selectively target resistant cancer cells by modifying their surface properties with tumor-targeting ligands or antibodies, enhancing drug uptake specifically by the cancer cells [97,98]. Additionally, the use of micelles helps to improve the solubility of poorly water-soluble drugs, further enhancing their bioavailability [99]. On the other hand, exosome-mimicking nanoparticles, which are bioengineered nanoparticles that mimic the natural exosomes used by cells for intercellular communication, have shown promise in overcoming MDR by facilitating intracellular drug delivery and bypassing the drug efflux pumps that cancer cells use to expel chemotherapy agents [100,101]. These nanoparticles can also be designed to modulate or inhibit the expression of MDR-related genes, effectively reversing resistance mechanisms [102,103]. Furthermore, the combination of these advanced materials with gene-silencing technologies or RNA interference approaches can inhibit the pathways responsible for resistance, thus restoring the sensitivity of cancer cells to chemotherapy [104,105]. By delivering drugs directly to the intracellular compartment and circumventing the drug resistance mechanisms, these novel nanocarriers significantly improve the efficacy of chemotherapy in resistant cancer types, potentially overcoming one of the most difficult challenges in modern oncology.

### 3.2. Chronic Disease Management

Chronic conditions such as diabetes, cardiovascular diseases, and neurological disorders require continuous management and strategic approaches. Advanced therapeutic materials have enabled the development of devices that ensure the controlled and sustained release of drugs, thereby improving patient compliance and safety.

#### 3.2.1. Diabetes Management

Advancements in injectable hydrogels and nanocarriers have brought transformative possibilities for diabetes management, particularly in providing sustained and controlled release of insulin and other antidiabetic agents [106,107,108]. Traditional insulin administration requires frequent injections, but these new systems offer prolonged drug release over extended periods, reducing the burden on patients and improving adherence to treatment [109,110]. Among the most notable innovations are glucose-sensitive materials, which are engineered to respond dynamically to changes in blood glucose levels [111,112]. These materials release insulin when glucose concentrations rise and cease release when glucose levels normalize, closely mimicking the body’s natural insulin regulation mechanism. By offering real-time responsiveness, these systems reduce the risk of both hyperglycemia and hypoglycemia, ensuring more stable blood sugar levels. They also decrease the frequency of insulin administration, increasing patient comfort and quality of life. Research into biodegradable polymers has enabled the development of nanocarriers that can encapsulate and release antidiabetic agents such as glucagon-like peptide-1 (GLP-1) analogs in response to glucose levels, offering personalized care options [113,114]. Additionally, innovations such as smart insulin patches are being explored, which can autonomously release insulin based on blood glucose fluctuations, providing even more personalized and patient-friendly solutions [115]. The combination of these advanced systems offers the potential for improved glycemic control with fewer side effects, ultimately leading to better disease management and better long-term outcomes for diabetic patients.

#### 3.2.2. Cardiovascular Disorders

The treatment of cardiovascular disorders, particularly post-angioplasty, has greatly benefited from the use of drug-eluting stents. These stents, typically made from biodegradable polymers, provide localized delivery of antiproliferative drugs to the affected blood vessels, preventing restenosis (re-narrowing of the arteries) [116,117]. The key advantage of these stents lies in their ability to release drugs precisely where needed, minimizing the exposure of other organs to the drugs and reducing systemic side effects. In the recent advancements, the focus has been on improving biocompatibility and reducing inflammation, which can occur during the healing process after stent insertion [118,119]. New stent coatings are being developed to enhance the integration of the stent with the vessel wall, reduce inflammatory reactions, and improve the endothelial healing process [116,120]. Researchers are working on improving the drug release kinetics of stents to ensure that drugs are delivered over the appropriate time period, balancing efficacy and reducing the risk of restenosis [121,122]. Furthermore, biodegradable drug-eluting stents have made it possible for the stent material to dissolve over time, avoiding the need for surgical removal and preventing long-term complications associated with permanent metallic stents [117,123]. Other advancements include the incorporation of polymers with stimuli-responsive properties, which release drugs in response to specific factors, such as pH changes or inflammatory markers, ensuring more targeted and effective treatment [124,125]. These innovations are helping to improve patient outcomes by reducing complications, enhancing the healing process, and offering long-term protection against restenosis, without the need for repeated interventions.

#### 3.2.3. Neurological Disorders

Treating neurological disorders such as Alzheimer’s disease and Parkinson’s disease remains an ongoing challenge due to the protective blood–brain barrier (BBB), which prevents most therapeutic agents from crossing into the brain. To overcome this challenge, innovative lipid-based nanoparticles and polymeric scaffolds are being explored as potential solutions for targeted drug delivery to the brain [126,127,128]. These delivery systems are designed to cross the BBB either by exploiting specific transport mechanisms or by modifying their surface properties to enhance permeability. Lipid nanoparticles, such as solid lipid nanoparticles (SLNs) or liposomes, have shown promise in delivering neuroprotective agents such as antioxidants, neurotrophic factors, and anti-inflammatory drugs directly to the brain [129,130,131]. These systems can also be engineered to release their payloads in a controlled manner, which improves drug efficacy and minimizes side effects. Research has also focused on the development of polymeric nanoparticles and nanogels that can encapsulate multiple therapeutic agents and release them in a targeted, sustained manner, ensuring that higher concentrations of the drug reach the intended site of action within the brain [132,133]. Advances in polymeric scaffolds have enabled the development of biomaterials that can not only carry drugs to the brain but also serve as structural support for damaged tissues, aiding in neuroregeneration [134,135]. For example, hydrogel-based systems have been developed to deliver nerve growth factors to promote the regeneration of neurons in conditions such as Parkinson’s disease [136,137,138]. These systems are designed to interact with specific regions of the brain that are affected by disease, improving the delivery of therapeutic agents and providing more personalized, effective treatment options. Moreover, strategies such as nanoparticle-functionalized antibodies and aptamers are being investigated to enhance targeting specificity, ensuring that drugs reach only the affected areas, thereby minimizing off-target effects [139,140]. With these advancements, it is now possible to provide more precise, controlled, and effective treatments for neurological disorders, opening new doors for better patient care, and potentially slowing down or modifying the course of these debilitating diseases.

### 3.3. Vaccine Delivery

Vaccines are necessary to prevent infectious diseases and limit the scope of pandemics. This means that the success of vaccines depends on achieving strong and sustained immune responses and that new materials have helped to overcome delivery problems that were encountered with conventional methods.

#### 3.3.1. Lipid Nanoparticles (LNPs)

Lipid nanoparticles (LNPs) have become a cornerstone in modern vaccine technology, particularly with the rapid development of mRNA-based vaccines, such as the Pfizer-BioNTech and Moderna COVID-19 vaccines [141,142]. LNPs are nanoscale particles composed primarily of lipids that are designed to encapsulate and protect fragile molecules such as mRNA, ensuring their stability during storage and transportation [143]. In the context of mRNA vaccines, LNPs serve as the delivery vehicle that shields the mRNA from enzymatic degradation and facilitates its delivery into host cells [144].

The success of LNPs in mRNA vaccines is largely attributed to their ability to effectively interact with cell membranes [145]. When administered, LNPs are taken up by cells through endocytosis, where the mRNA is then released into the cytoplasm [146]. The LNPs’ lipid composition is crucial for this process, as the lipid bilayer can fuse with the cell membrane, allowing the mRNA to be directly delivered into the cells without triggering excessive immune responses or causing toxicity [147,148]. Once inside the cell, the mRNA is translated into the protein of interest, such as the spike protein in the case of the COVID-19 vaccines, triggering an immune response that prepares the body to recognize and fight the virus if it is encountered again [149].

One of the key advantages of LNPs in vaccine delivery is their ability to induce strong immune activation [150,151]. They enhance the uptake of mRNA by dendritic cells and other antigen-presenting cells, which are responsible for initiating the immune response. This leads to the production of both neutralizing antibodies and T-cell responses, both of which are crucial for protecting against viral infections [150,152,153]. Additionally, LNPs have shown promise in targeting specific tissues or organs, increasing the efficiency of vaccine delivery while potentially reducing side effects or systemic toxicity [154,155].

LNPs are also highly versatile, allowing for customization of their lipid components to optimize the release profile, stability, and biocompatibility of the mRNA payload [156]. Variations in the lipid composition such as the inclusion of cationic lipids (positively charged lipids) or helper lipids (which assist in the formulation’s stability) can be used to modulate the size and charge of the nanoparticles, improving their effectiveness and reducing potential immune reactions [147,148]. Furthermore, LNPs have been designed to be scalable and mass-producible, which is essential for meeting the global demand for vaccines, especially in times of urgent health crises such as the COVID-19 pandemic.

Looking to the future, LNPs are expected to play a pivotal role in the development of next-generation vaccines. Their ability to encapsulate different types of genetic material, such as DNA, mRNA, or even viral vectors, means that LNPs are not limited to a single type of vaccine but can be used across a broad spectrum of diseases, including influenza, Zika virus, HIV, and cancer vaccines. As viruses evolve, particularly rapidly mutating viruses such as the SARS-CoV-2 virus, LNP-based platforms offer the flexibility to quickly modify the mRNA payloads, facilitating faster development of updated vaccines without needing to redesign the entire delivery system.

Additionally, LNPs are being explored for their potential use in therapeutic vaccine development, where they can deliver mRNA-based treatments for various diseases, including cancers, genetic disorders, and autoimmune diseases [157,158]. The advancements in lipid nanoparticle technology will likely drive the next wave of vaccine innovation, providing a universal platform for the rapid response to emerging infectious diseases and the potential to tackle more complex diseases. With ongoing improvements in formulation, stability, and delivery mechanisms, LNPs are set to remain a critical technology in vaccine development, offering enhanced immunogenicity, efficacy, and safety profiles for a wide range of global health challenges.

#### 3.3.2. Polymeric Scaffolds

Polymeric scaffolds are gaining attention as an innovative strategy for vaccine delivery, offering controlled and sustained release of antigens to improve immune responses [159,160]. These biocompatible and biodegradable materials can be designed to mimic the extracellular matrix (ECM), providing a supportive environment for immune cell interaction and enhancing antigen presentation. Polymeric scaffolds can be engineered to encapsulate vaccines, enabling targeted delivery and release at specific sites, which can enhance vaccine efficacy and reduce side effects [161]. Additionally, these scaffolds can be used to create depot systems that release vaccines over an extended period, offering long-lasting protection with fewer doses [162]. Commonly used materials for scaffold fabrication include natural polymers such as collagen and chitosan and synthetic polymers, including PLGA and PEG, which offer tunable properties for optimal vaccine delivery [163]. The ability to incorporate bioactive molecules, such as adjuvants or immune-modulating agents, into polymeric scaffolds further boosts their potential for improving vaccine performance, particularly in overcoming challenges such as the need for cold storage and enhancing responses in immunocompromised populations [164,165]. As research continues, polymeric scaffolds hold the potential to revolutionize vaccine delivery systems, making them more effective, accessible, and long-lasting.

#### 3.3.3. Microneedle Patches

Microneedle technology represents a groundbreaking approach in vaccine delivery, offering significant advantages over traditional methods, particularly in low-resource settings [166,167]. These patches consist of arrays of tiny, painless needles, typically ranging from 50 to 900 μm in length, which are designed to painlessly penetrate the outermost layer of the skin (the epidermis) to deliver the vaccine [168,169]. One of the key benefits of microneedles is their ease of use, patients can apply the patches themselves, reducing the need for trained healthcare professionals and facilitating widespread distribution. This self-administration capability enhances adherence to vaccination schedules, particularly in regions where access to medical facilities is limited. Moreover, microneedle patches do not require the cold chain storage that most traditional vaccines demand, making them ideal for use in resource-poor or remote areas, where refrigeration is often unavailable. The ability to store and transport vaccines without refrigeration greatly simplifies logistical challenges and increases vaccine accessibility. Additionally, the microneedles enhance the stability of the antigens delivered, protecting them from degradation, and ensuring that the immune system receives the optimal dose for effective activation [170,171,172]. By delivering the vaccine directly to the skin’s immune-rich layers, microneedles stimulate a stronger immune response, as the skin contains a high density of dendritic cells that are key to initiating immune reactions [171,173]. This technology not only offers a minimally invasive and pain-free alternative to traditional needles, but it also improves vaccine efficacy and patient compliance, thereby playing a crucial role in achieving higher vaccination coverage, particularly in underserved regions. As microneedle technology continues to advance, it holds the potential to revolutionize vaccine delivery, making immunization more efficient, accessible, and scalable across diverse global populations.

#### 3.3.4. Adjuvant Systems

Recent advancements in adjuvant systems, particularly those utilizing polymeric nanoparticles, have significantly enhanced the effectiveness and efficiency of vaccines. These novel adjuvants work by improving the immunogenicity of vaccines, stimulating a stronger and longer-lasting immune response [174,175]. One of the key benefits of polymeric nanoparticle-based adjuvants is their ability to reduce the antigen load required for an effective immune response, which helps in lowering production costs and increasing vaccine availability, a crucial factor in global vaccination campaigns [175]. These nanoparticles are designed to facilitate the sustained release of antigens over time, allowing for prolonged immune activation. This extended release enhances the duration of protection against infectious diseases by maintaining the immune system’s alertness and ensuring that the body is constantly exposed to the antigen, thus boosting both humoral and cell-mediated immunity [176]. Furthermore, these polymeric nanoparticles can also act as carriers for other immunostimulatory molecules, ensuring that the immune response is not only strong but also well-targeted to the specific pathogen [177,178]. By improving bioavailability, stability, and the controlled release of vaccine components, polymeric nanoparticle adjuvants help to optimize vaccine efficacy, enabling more efficient and cost-effective immunization strategies. As a result, these systems hold great promise in addressing global health challenges by making vaccines more accessible and improving vaccine-induced protection, especially in regions with limited healthcare resources.

**Figure 3:**
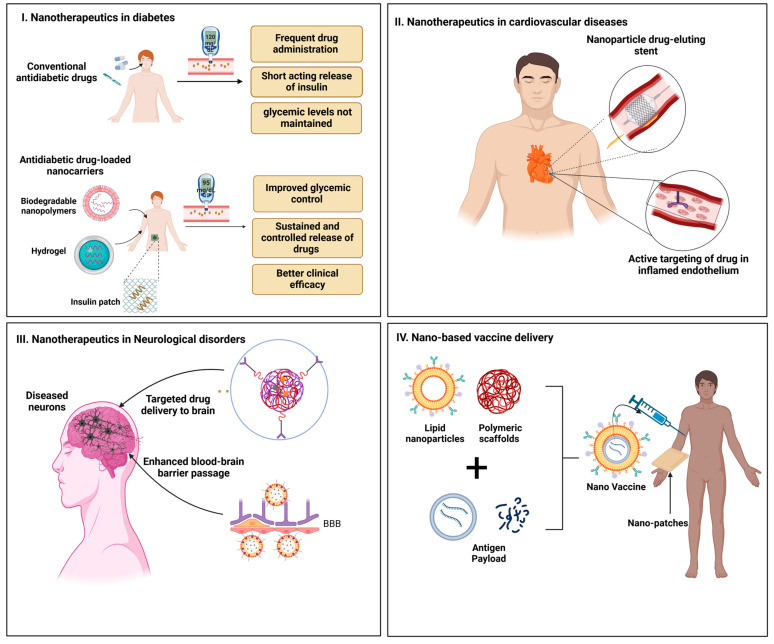
Applications of nanotherapeutics in diverse diseases. This schematic illustrates the applications of nanotherapeutics in four distinct disease areas: (**I**) diabetes, showcasing the advantages of nanocarriers for sustained insulin delivery; (**II**) cardiovascular diseases, highlighting the use of nanoparticle drug-eluting stents and targeted drug delivery; (**III**) neurological disorders, demonstrating enhanced drug delivery across the blood–brain barrier; and (**IV**) nano-based vaccine delivery, featuring lipid and polymeric nanoparticles for antigen delivery and nano-patches for administration. The figure emphasizes the potential of nanotechnology to improve treatment efficacy and patient compliance across various medical conditions.

The application of advanced materials in drug delivery systems has expanded into therapeutic applications and can be cited in oncology, chronic disease management, and vaccine delivery (Figure 3). These materials provide answers to some of the most enduring questions in a way that allows for more targeted, efficient, and patient-friendly therapies. Nevertheless, their fullest usefulness can only be achieved by overcoming the translational issues of biocompatibility, scalability, and regulatory issues. As research on these technologies improves and evolves, advanced materials will become a key driver of pharmaceutical innovation and continue to improve global healthcare.

## 4. Translational Challenges

The application of advanced materials in drug delivery has the transformative potential, but there are several challenges that need to be overcome for these technologies to gain widespread clinical adoption. The most pressing concerns are biocompatibility and safety, scalability in manufacturing, and the complex regulatory landscape. These factors are therefore critical in helping to move ideas born in the laboratory to the clinic.

### 4.1. Biocompatibility and Safety

The safety and biocompatibility of advanced materials over the long-term remain an issue of translational concern. While these materials exhibit excellent performance in drug delivery, they can interact with the biological system in a way that is not well understood and may present unwanted effects, such as immune response, toxicity, and tissue retention.

#### 4.1.1. Immune Reactions

This is particularly the case for nanoparticles, which are made of non-biodegradable or inorganic materials and can react with the immune system, triggering it to attack them or to activate the complement system. This can lead to inflammation, hypersensitivity, or other issues that affect the healing of the patient. To reduce immunogenicity and increase the circulation time, nanoparticles are surface modified with polyethylene glycol (PEG). However, the use of biodegradable polymers, including PLGA, is also emerging as a way to limit immune activation and enhance biocompatibility.

#### 4.1.2. Degradation Products

The advanced materials can also break down, whether it is through enzymatic action or environmental stress, to form products that can accumulate in the body or interfere with its function. For instance, certain polymers can degrade to produce acidic compounds that may cause inflammation or toxicity at the local site. This makes it important to study the kinetics of biodegradation and clearance in preclinical studies to ensure that these compounds do not have harmful effects in the long run. In vivo, material degradation and biodistribution can be monitored through advanced imaging techniques and bioassays, which help in evaluating the safety of these materials.

#### 4.1.3. Chronic Exposure Risks

For instances where these materials are administered frequently or in large quantities, whether it is in the management of chronic conditions or in the treatment of cancer, it is crucial to consider the cumulative exposure to these materials. The accumulation of nanoparticles in the liver, spleen, and kidneys has been a subject of concern with regards to their toxicity, underscoring the importance of developing nanoparticles that can be easily excreted from the body.

Although the explanation is brief, it effectively conveys the idea that, while the concept of digital assets is intuitive, there are precise definitions for each type of asset (cryptocurrencies, security-based digital assets), and understanding these distinctions is crucial for navigation in the evolving regulatory landscape. By recognizing the differences between these assets, investors, companies, and regulators can make informed decisions and avoid potential pitfalls, ultimately promoting stability and innovation in the digital asset space.

### 4.2. Scalability and Manufacturing

The transition from laboratory-scale synthesis to large-scale industrial manufacturing is a critical step in the commercialization of advanced nanomaterials. However, these complex materials pose significant challenges related to cost, consistency, quality control, and long-term stability.

#### 4.2.1. Cost of Production

The production of advanced materials such as nanoparticles and bioresponsive polymers often requires specialized equipment, rare reagents, and time-intensive synthesis processes, making large-scale production costly and resource-intensive. These factors limit material availability and scalability. To address this, researchers are exploring cost-effective synthetic approaches, such as green chemistry, biomimetic synthesis, and the use of abundant, renewable raw materials. Additionally, automation and process optimization are being integrated into manufacturing workflows to reduce labor costs, minimize waste, and enhance production efficiency.

#### 4.2.2. Batch-to-Batch Variability

Ensuring batch-to-batch consistency is crucial for regulatory approval, clinical applications, and therapeutic reliability. Key physicochemical properties such as nanoparticle size, surface charge, drug-loading efficiency, and release kinetics directly impact drug efficacy and safety. Variability in these characteristics can compromise therapeutic outcomes, leading to challenges in reproducibility and standardization. To mitigate this, sophisticated analytical techniques such as high-performance liquid chromatography (HPLC), dynamic light scattering (DLS), electron microscopy, and zeta potential measurements are employed for real-time monitoring and stringent quality control. Establishing robust quality control checkpoints throughout the production process ensures greater reproducibility and regulatory compliance.

#### 4.2.3. Stability of Colloidal Suspensions

A significant challenge in nanocarrier manufacturing is maintaining the long-term stability of colloidal suspensions, as their properties can be affected by storage conditions, aggregation, sedimentation, and chemical degradation over time. Factors such as pH, ionic strength, temperature, and exposure to light can alter particle size, surface charge, and drug release profiles, ultimately impacting the shelf life and therapeutic performance of the nanocarrier formulations. Strategies to enhance colloidal stability include surface modification with stabilizing agents (e.g., PEGylation), optimization of storage buffers, lyophilization (freeze-drying), and encapsulation within protective matrices. Additionally, real-time stability testing under accelerated aging conditions is being integrated into manufacturing pipelines to predict long-term performance and ensure regulatory compliance.

#### 4.2.4. Scalable Manufacturing Technologies

To meet the increasing demand for commercial-scale production, researchers are investigating high-throughput, scalable manufacturing technologies such as microfluidics, continuous flow synthesis, and electrospray techniques. These methods enable precise control over nanoparticle size, shape, and surface properties, ensuring uniformity across large batches. Microfluidic systems, in particular, allow for rapid, controlled nanoparticle synthesis with minimal waste, making them a promising scalable alternative for industrial applications. Additionally, advancements in 3D printing and self-assembly techniques are being explored for high-precision fabrication of nanocarrier-based drug delivery systems.

By addressing challenges related to cost, batch variability, colloidal stability, and scalability, researchers are working toward standardized, cost-efficient, and high-quality nanocarrier manufacturing, bringing next-generation nanomedicines closer to clinical and commercial realization.

### 4.3. Regulatory Considerations

The regulatory landscape for advanced materials in drug delivery remains complex and still under development. These materials, which include cutting-edge nanocarriers, hydrogels, bioresponsive polymers, and other innovative technologies, pose unique challenges for regulatory bodies. Traditional regulatory frameworks, designed for more conventional drug delivery systems, are not fully equipped to assess these novel materials comprehensively. Regulatory agencies such as the U.S. Food and Drug Administration (FDA—https://www.fda.gov/files/drugs/published/Drug-Products--Including-Biological-Products--that-Contain-Nanomaterials---Guidance-for-Industry.pdf accessed on 8 March 2025) and the European Medicines Agency (EMA—https://www.ema.europa.eu/en/human-regulatory-overview/research-development/scientific-guidelines accessed on 8 March 2025) are increasingly focused on creating guidelines for these emerging technologies. However, a more standardized, harmonized, and adaptable regulatory framework is required to facilitate the approval of advanced materials in drug delivery and accelerate their market introduction.

#### 4.3.1. Lack of Established Guidelines

The advent of nanotechnology, bioresponsive polymers, and other innovative materials has led to the development of entirely new classes of materials that were not considered during the creation of existing regulatory guidelines. These new materials require a more specialized regulatory approach due to their distinct properties, such as nano-scale size, surface modification, and bioactivity, which differ significantly from conventional drug carriers. As a result, regulatory agencies are often uncertain about how to evaluate them, particularly regarding their long-term safety and impact on human health. While the FDA and EMA have started developing guidelines for evaluating nanomedicines and other advanced materials, the lack of clear, standardized international frameworks continues to create confusion, delay market entry, and increase the risk of inconsistent regulatory decisions. Additionally, there is a pressing need for guidelines that address the lifecycle of these materials, from initial research and preclinical trials to clinical application and post-market surveillance.

#### 4.3.2. Clinical Validation

For advanced materials to be approved for clinical use, comprehensive preclinical and clinical trials are essential to demonstrate their safety, efficacy, and performance. These trials must include detailed assessments of biocompatibility, biodistribution, toxicity, and potential immunogenicity, among other factors. In the case of nanocarriers, for instance, it is crucial to understand how the material interacts with the body, including the liver, spleen, and kidneys, and how it may accumulate in tissues over time. In addition to traditional testing, the evolving nature of advanced materials means that trials must also consider new variables, such as response to environmental stimuli (e.g., pH or temperature changes). To ensure the timely progression of these trials, collaboration among academic researchers, pharmaceutical industry professionals, and regulatory bodies is necessary. Adaptive trial designs are being explored as a potential solution, allowing for modifications based on early results. This flexibility can make trials more efficient, cost-effective, and responsive to emerging data, ultimately speeding up the approval process without compromising patient safety.

#### 4.3.3. Material Characterization Standards

Material characterization is a vital component of the regulatory approval process, particularly for advanced materials used in drug delivery. Regulators require detailed information on the size, shape, surface chemistry, mechanical properties, and biological activity of materials to assess their suitability for clinical applications. For example, nanocarriers must be evaluated for their size distribution, surface charge, and stability in biological environments. However, the methods used to characterize these materials vary widely across different research settings, and standardized protocols are still being developed. The lack of harmonized characterization methods can result in inconsistent data, leading to regulatory uncertainties. Establishing standardized and internationally recognized characterization methods will simplify the approval process and reduce the risk of delays. Creating global databases that include material properties and their corresponding performance data could provide valuable resources for regulators, clinicians, and researchers. Furthermore, the development of new techniques to assess the long-term effects of these materials in the body such as their biodegradability, metabolic by-products, and long-term safety will be critical to advancing their clinical application.

### 4.4. Integration and Future Perspectives

The successful integration of advanced materials into clinical practice hinges on overcoming a series of technical, regulatory, and logistical challenges. While these materials hold enormous potential to revolutionize drug delivery, they must meet rigorous safety, efficacy, and manufacturing standards to ensure their widespread use. For instance, biocompatibility is a fundamental requirement, as the materials must be non-toxic, non-immunogenic, and capable of being cleared from the body without adverse effects. Additionally, the manufacturing processes for advanced materials must be scalable, reproducible, and cost-effective to meet the demands of large-scale production. The regulatory framework needs to support these processes by offering clear, scientifically validated pathways for approval that can be uniformly applied across jurisdictions.

Collaboration among researchers, manufacturers, and policymakers is critical to overcoming these hurdles. Researchers can provide the scientific evidence needed to support regulatory decisions, while manufacturers can develop the technologies to scale up production. Policymakers, in turn, must establish regulatory frameworks that are flexible enough to accommodate the rapid pace of innovation in the field while maintaining high safety standards. This collaborative effort will be essential in ensuring that advanced materials not only reach the clinical stage but also have the desired impact on patient care. As these materials continue to evolve, their role in personalized medicine and drug delivery will only expand. Nanocarriers, hydrogels, and bioresponsive polymers are expected to become integral components of targeted therapies, allowing for more precise, controlled, and sustained delivery of therapeutic agents. The convergence of advanced materials with emerging technologies such as artificial intelligence (AI) and machine learning (ML) will further enhance their capabilities. AI and ML can optimize material design, predict clinical outcomes, and enable real-time adjustments to treatment regimens, ultimately leading to more personalized and effective therapies.

**Figure 4:**
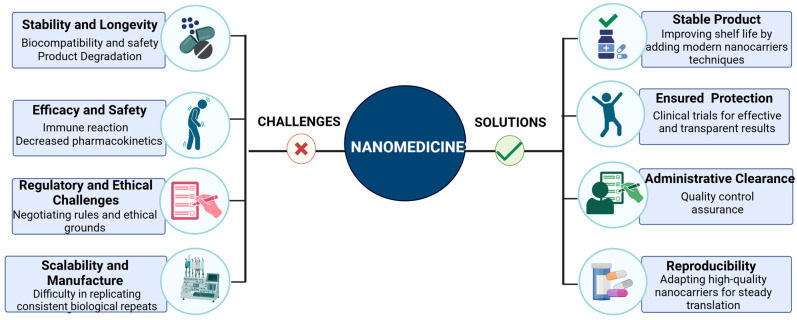
Navigating challenges and solutions in nanomedicine. This schematic outlines the key challenges encountered in the development and translation of nanomedicine, along with the corresponding solutions. The figure is divided into two sections: “Challenges” on the left, encompassing aspects such as stability, efficacy, regulatory hurdles, and scalability; and “Solutions” on the right, highlighting strategies for achieving stable products, ensured protection, administrative clearance, and reproducibility. The central element, “Nanomedicine”, is depicted as a pivotal point, emphasizing its role in addressing these challenges through innovative solutions.

In the future, advanced materials may become increasingly important in addressing the needs of underserved populations and rare diseases. For example, they could be used to develop more efficient vaccine delivery systems or improve treatments for conditions such as cancer, diabetes, and neurological diseases. As research progresses and technologies mature, advanced materials will continue to provide innovative solutions to unmet medical needs, making treatments more effective, accessible, and equitable on a global scale. However, the full potential of these materials can only be realized if these challenges are addressed through collaborative efforts, ensuring that the benefits of these technologies are available to patients worldwide (Figure 4).

## 5. Future Directions

**Figure 5:**
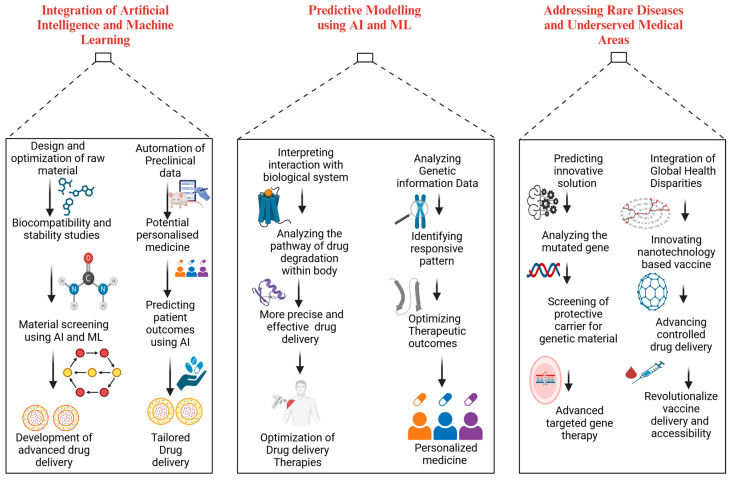
Applications of artificial intelligence and machine learning in drug development and healthcare. This schematic illustrates the diverse applications of AI and ML in various stages of drug development and healthcare, categorized into three key areas: (1) integration of AI and ML for drug design and optimization, (2) predictive modeling for personalized medicine and improved outcomes, and (3) addressing rare diseases and underserved medical areas. Each section highlights specific applications with corresponding icons, demonstrating the potential of AI and ML to revolutionize drug discovery, delivery, and patient care.

Advanced materials for drug delivery are likely to be integrated with emerging technologies and expand into new therapeutic areas to meet the increasing complexity of healthcare needs. These materials have the potential to reduce the complexity of the pharmaceutical industry in the future by overcoming the existing limitations and fostering innovation. Using artificial intelligence (AI), addressing rare diseases, and exploring underserved medical areas, advanced materials can further develop drug delivery systems to improve patient outcomes globally (Figure 5).

### 5.1. Integration of Artificial Intelligence and Machine Learning

The integration of artificial intelligence (AI) and machine learning (ML) is poised to significantly transform the development of advanced materials for drug delivery by harnessing computational power to analyze vast datasets, uncover patterns, and predict outcomes more efficiently. These technologies can optimize the design and synthesis of new materials by predicting their properties, such as biocompatibility, stability, and drug release profiles, even before they are synthesized in the lab. AI and ML algorithms can accelerate material screening, reducing time and cost by identifying the most promising candidates for further development. Additionally, they can streamline the characterization process by automating the analysis of complex data from preclinical trials, such as biological responses and toxicity assessments. These tools also hold great potential in personalized medicine, where AI and ML can be used to tailor drug delivery systems to individual patients based on their unique genetic makeup, disease conditions, and treatment responses. Furthermore, AI and ML can enhance clinical trial designs by enabling adaptive trials, predicting patient outcomes, and optimizing drug delivery regimens. In the future, these technologies will likely play a pivotal role in advancing precision medicine, enabling the creation of highly targeted, efficient, and safe drug delivery systems that can address unmet medical needs across various diseases. By integrating AI and ML, the entire lifecycle of drug delivery materials, from discovery and design to clinical applications, can be made more efficient, effective, and scalable, ultimately improving patient care and outcomes.

#### 5.1.1. Predictive Modeling

Predictive modeling powered by artificial intelligence (AI) offers a groundbreaking approach to simulating how materials interact with biological systems, significantly reducing the reliance on time-intensive and costly experimental studies. For instance, AI algorithms can predict the interaction of nanocarriers with cellular receptors, providing valuable insights into how these materials may trigger immune responses or degrade within the body. By accurately forecasting these interactions, researchers can expedite the material development process, enhancing both the speed and efficiency of selecting materials with optimal biocompatibility and performance. Furthermore, AI-driven platforms can model drug release kinetics, predicting how a drug will be released from its delivery system over time, based on various factors such as material properties, environmental conditions, and formulation characteristics. This enables the design of more precise and effective drug delivery systems, tailored to release therapeutic agents at the right dose and at the optimal time, thereby improving therapeutic outcomes. Predictive modeling also allows for the identification of potential challenges and the optimization of drug delivery strategies before conducting extensive in vivo or clinical trials, accelerating the development of safer and more effective therapies.

#### 5.1.2. Personalized Medicine

Personalized medicine, powered by machine learning (ML), is revolutionizing the way healthcare providers design and deliver treatments tailored to the unique needs of each patient. By analyzing a vast array of data, including genetic information, metabolic profiles, environmental exposures, and lifestyle factors, ML algorithms can identify patterns and predict how individual patients will respond to different drugs, dosages, and delivery methods. This enables the creation of highly customized drug delivery systems that optimize therapeutic outcomes while minimizing side effects. For instance, ML can determine the most effective drug formulation based on a patient’s genetic makeup, ensuring that treatments are specifically targeted to their biological needs [179,180,181]. Additionally, wearable health devices and continuous monitoring tools can provide real-time data that help adjust the drug release profiles in adaptive delivery systems. This responsiveness allows for treatments to evolve in sync with a patient’s changing health status, offering a level of precision that traditional medicine cannot match. Furthermore, ML-driven models can assist in early diagnosis and preventive care by predicting disease progression and suggesting personalized interventions before symptoms even arise. In oncology, for example, ML can be used to identify specific genetic mutations in tumors, leading to the development of targeted therapies that address the root causes of the disease, rather than just its symptoms. By integrating genetic, clinical, and environmental data, personalized medicine aims to provide more effective, safer, and cost-efficient treatments, ultimately improving patient outcomes and paving the way for a future where healthcare is fully customized for every individual.

### 5.2. Expansion to Rare Diseases

Advanced materials provide new and exciting possibilities for addressing the unmet needs of patients with rare and underserved diseases. Customized delivery platforms have the potential to revolutionize the treatment options for diseases that currently have limited or no effective treatment.

#### 5.2.1. Gene and Cell Therapy

Rare genetic disorders call for innovative solutions, and current advanced materials including lipid nanoparticles and hydrogels are used in the delivery of gene-editing tools such as CRISPR-Cas9 and RNA-based therapies. These materials serve as protective carriers of genetic materials and ensure their efficient internalization by cells, which can then use them to modify their genome and correct the mutation causing the disease. In cell therapy, hydrogels are investigated as a scaffold to support the survival, proliferation, and integration of cells within the target tissue, such as in muscular dystrophy and cystic fibrosis.

#### 5.2.2. Orphan Drug Development

Orphan drugs are treatments for rare diseases, such as cystic fibrosis, Lou Gehrig’s disease, and Tourette’s syndrome. These drugs are developed and marketed for small patient populations, often presenting high development and commercial risks. The versatility of advanced materials can help simplify the preparation of targeted delivery systems, which may help reduce the costs of development and enhance the therapy outcomes. For instance, nanoparticles can be designed to target drugs to specific tissues and thus reduce the systemic drug exposure and side effects. These innovations are encouraging investors to spend on orphan drug development, making much-needed therapies available to patients with rare diseases.

### 5.3. Exploration of Underserved Areas

Advanced materials have the potential to address the challenges of emerging infectious diseases, aging populations, and global health disparities due to their versatility and adaptability. These materials are very useful in increasing the accessibility of healthcare and improving therapeutic outcomes in underserved areas, where healthcare infrastructure may be limited.

#### 5.3.1. Global Vaccine Accessibility

The COVID-19 pandemic has shown the role of advanced materials, including lipid nanoparticles, in vaccine development. Future innovations, including microneedle patches and thermally stable formulations, could revolutionize vaccine delivery in low-resource settings. For example, microneedle patches are an example of painless, self-administered vaccination that does not require cold chain storage. These advances improve vaccine availability in remote areas and reduce barriers to immunization against infectious diseases.

#### 5.3.2. Aging-Related Therapies

The increasing population of elderly people with chronic conditions such as osteoporosis, arthritis, and neurodegenerative diseases poses unique healthcare challenges. Advanced materials for controlled drug release provide targeted and sustained therapeutic effects, decreasing the frequency of dosing. For instance, hydrogels are being developed to deliver growth factors and anti-inflammatory drugs to enhance cartilage repair in osteoarthritis. Likewise, lipid-based nanoparticles are being investigated for delivering neuroprotective agents. The future of advanced materials in drug delivery lies in exploring new therapeutic agents for the treatment of brain diseases such as Alzheimer’s and Parkinson’s.

### 5.4. Future Prospects and the Integration of Closely Linked Areas

The technological advancements in AI and ML are poised to revolutionize material design and the personalization of therapies. These cutting-edge technologies hold significant promise in enabling the development of innovative treatments, particularly for rare diseases and underserved areas that have traditionally lacked adequate medical solutions. As AI and ML continue to evolve, fostering collaboration among researchers, clinicians, and industry stakeholders will be essential to address the remaining challenges in this field. Such partnerships can help optimize the application of advanced materials and accelerate their integration into clinical practice. By leveraging these opportunities, advanced materials have the potential to transform healthcare, making treatments more effective, widely accessible, and equitable on a global scale.

## 6. Conclusions

The integration of advanced materials into drug delivery systems has become the new normal in pharmaceutical science to address the complexity of modern medicine. These innovations have used technologies such as nanocarriers, hydrogels, and bioresponsive polymers to enable precision, efficiency, and superior therapeutic outcomes. Advanced materials have revolutionized drug delivery by improving upon conventional systems, and their application in various fields, including oncology, chronic disease management, and vaccine delivery, has been remarkable.

Although these materials show great promise, there are still issues that need to be overcome for them to be widely used in clinical practice. Ongoing research is needed to fully understand the biocompatibility, long-term safety, and degradation products of these materials. Additionally, concerns about scalability in manufacturing and regulatory standards are valid. These issues cannot be resolved until researchers, clinicians, and policymakers put their heads together to guarantee that these materials are safe, effective, and available to everyone.

The use of advanced materials in drug delivery is closely linked to the development of new technologies and the application of therapies. The integration of artificial intelligence and machine learning is expected to revolutionize material design by allowing predictive modeling and personalized medicine that meets the needs of individual patients. These tools can be used to fine-tune material properties, lower the time it takes to develop new materials, and develop systems that can adjust in real-time based on a patient’s input.

In addition, these materials have the ability to address unmet medical needs in rare diseases and other underserved populations. Their use in gene and cell therapy and orphan drugs points to their potential to change treatments for diseases with limited treatment options. In addition, their use in global vaccine accessibility and aging-related therapies indicates their capability to address the changing healthcare needs of a diverse and aging population.

This article clearly highlights the potential of advanced materials to transform the pharmaceutical sector. With the help of these technologies, existing barriers can be overcome, and drug delivery can be improved to make treatments more effective, safe, and available to all. Advanced materials have the capacity to be utilized to their fullest potential through collaboration among academia, industry, and regulatory agencies to enhance patient care and improve global health outcomes. The role of advanced materials in drug delivery is one of dynamic development: a future in which therapies are not only enhanced but also universal and targeted to the needs of each patient.
