# Correction: El-Tanani et al. Revolutionizing Drug Delivery: The Impact of Advanced Materials Science and Technology on Precision Medicine. *Pharmaceutics* 2025, *17*, 375

**DOI:** 10.3390/pharmaceutics17091165

**Published:** 2025-09-05

**Authors:** Mohamed El-Tanani, Shakta Mani Satyam, Syed Arman Rabbani, Yahia El-Tanani, Alaa A. A. Aljabali, Ibrahim Al Faouri, Abdul Rehman

**Affiliations:** 1 RAK College of Pharmacy, Ras Al Khaimah Medical and Health Sciences University, Ras Al Khaimah P.O. Box 11172, United Arab Emirates; 2 Department of Pharmacology, RAK College of Medical Sciences, Ras Al Khaimah Medical and Health Sciences University, Ras Al Khaimah P.O. Box 11172, United Arab Emirates; 3 Royal Cornwall Hospital NHS Trust, Truro TR1 3LJ, UK; 4 Department of Pharmaceutics and Pharmaceutical Technology, Faculty of Pharmacy, Yarmouk University, Irbid 21163, Jordan; 5 RAK College of Nursing, Ras Al Khaimah Medical and Health Sciences University, Ras Al Khaimah P.O. Box 11172, United Arab Emirates; 6 Department of Pathology, RAK College of Medical Sciences, Ras Al Khaimah Medical and Health Sciences University, Ras Al Khaimah P.O. Box 11172, United Arab Emirates

## Reference Correction

After the publication of the review article [1], one of the referenced manuscripts was found to have been retracted. To enhance the quality of the manuscript, we have decided to replace the previous reference number 50 (Dristant et al.) from the original manuscript with the new reference (Sharma et al.) at the same position in the reference list.

Original reference number 50: Dristant, U.; Mukherjee, K.; Saha, S.; Maity, D. An overview of polymeric nanoparticles-based drug delivery system in cancer treatment. *Technol. Cancer Res. Treat.* **2023**, *22*, 15330338231152083.

New reference number 50: Sharma, S.; Sudhakara, P.; Singh, J.; Ilyas, R.A.; Asyraf, M.R.M.; Razman, M.R. Critical review of biodegradable and bioactive polymer composites for bone tissue engineering and drug delivery applications. *Polymers* **2021**, *13*, 2623.

The overall numbering and order of the references remain unchanged. The authors state that the scientific conclusions are unaffected. This correction was approved by the Academic Editor. The original publication has also been updated.
